# Neural correlates of economic game playing

**DOI:** 10.1098/rstb.2008.0165

**Published:** 2008-10-01

**Authors:** Frank Krueger, Jordan Grafman, Kevin McCabe

**Affiliations:** 1 Cognitive Neuroscience Section, National Institute of Neurological Disorders and Stroke, National Institutes of HealthBethesda, MD 20892-1440, USA; 2 The Center for the Study of Neuroeconomics, George Mason University4400 University Drive, MSN: 1G3, Fairfax, VA 22030, USA

**Keywords:** neuroeconomics, game theory, trust, reward, social, functional magnetic resonance imaging, oxytocin

## Abstract

The theory of games provides a mathematical formalization of strategic choices, which have been studied in both economics and neuroscience, and more recently has become the focus of neuroeconomics experiments with human and non-human actors. This paper reviews the results from a number of game experiments that establish a unitary system for forming subjective expected utility maps in the brain, and acting on these maps to produce choices. Social situations require the brain to build an understanding of the other person using neuronal mechanisms that share affective and intentional mental states. These systems allow subjects to better predict other players' choices, and allow them to modify their subjective utility maps to value pro-social strategies. New results for a trust game are presented, which show that the trust relationship includes systems common to both trusting and trustworthy behaviour, but they also show that the relative temporal positions of first and second players require computations unique to that role.

## 1. Introduction

Neuroeconomics brings together research in neuroscience and economics to better understand how actors make decisions by unifying mathematical constructs with behavioural measurements (McCabe 2002; Glimcher & Rustichini 2004; Camerer *et al*. 2005). Neuroeconomics research includes the study of social decision making or how actors make decisions when other actors are affected by the outcome, and/or other actors are also making decisions, and uses techniques such as functional magnetic resonance imaging (fMRI), positron emission topography (PET), transcranial magnetic stimulation (TMS) and pharmacological interventions. Game theory, formalized by von Neuman & Morgenstern (1944), has been very useful in helping to formulate experiments, and to interpret the decisions that actors make, and the neural signatures of these decisions in the brain. In studying decisions in an experiment, neuroeconomists are interested in recovering the neural computations/algorithm that subjects use to choose actions that ultimately result in outcomes. One of the goals of neuroeconomics is to provide a consistent biologically based connection between our functional and computational understanding of strategic choice.

Games can be analysed in different ways based on ways that strategies end up being chosen. Strategies may have evolved based on their fitness in the game ecology; they may be learnt through repeated interactions, or they may be deduced from the logic of the game. Each of these approaches is likely to involve a differential emphasis on the computations performed by a unitary neural system instantiated in the brain. For example, evolved strategies are likely to be driven more by pathways from sensory systems to expected utility maps and onto response systems, learned strategies will add a reinforcement learning strategy over both the probability that an event will occur and the contingent action that produces the highest expected reward, while deduced strategies are likely to involve more symbolic or abstract encodings and simulations of other people and will require evaluative attention to intermediate results in serial, what-if, computations. The human brain is likely to have evolved a functional capacity to choose strategies using neural systems operating at all three levels.

In this paper, we first briefly summarize the description and interpretation of neuroeconomics games under game theoretic aspects including experimental designs for the measurement and control of expected utility. Then, we describe experiments investigating primate behaviour in games against a computer and with other primates, before we review the literature on human economic game playing and its neural correlates. Finally, we present new fMRI findings regarding brain regions particularly involved in trust and reciprocity during economic exchange.

### (a) Describing and interpreting neuroeconomics games

The games that have been the focus of study so far by neuroeconomics are all two-actor games that are either competitive games, such as matching pennies game (MPG), rock–scissor–paper game (RSPG) and inspection game (ISG), or cooperative games, such as Prisoner's Dilemma game (PDG), equal split game (ESG), ultimatum game (UG), dictator game (DG), investment game (IG) and trust game (TG). In the following, we look at both how these games are formally defined and how game theory analyses these games.

The MPG, RSPG, ISG and PDG in figure 1 are presented as strategic form games; each player has to *simultaneously* make a choice from a set of choices. In particular, player 1 must choose a row from the set *S*_1_={*r*^1^, …, *r*^*n*^}, where *n* is the number of rows in the matrix. Call this choice player 1's strategy or denote it *s*_1_∈*S*_1_. At the same time, player 2 must choose a column from the set *S*_2_={*c*^1^, …, *c*^*m*^}, where *m* is the number of columns in the matrix. Call this choice player 2's strategy or denote it *s*_2_∈*S*_2_. The pair of strategies (*s*_1_, *s*_2_) is called a pure strategy profile. Strategy profiles determine a pay-off or utility for each player, denoted *U*_1_(*s*_1_, *s*_2_) and *U*_2_(*s*_1_, *s*_2_). For example, in the PDG (figure 1*d*), the rows and columns have been labelled cooperate and defect and the pay-offs are displayed in the matrix so that *U*_1_ (cooperate, defect)=0 and *U*_2_ (cooperate, defect)=3.

In strictly competitive games, also known as zero-sum games, each outcome has the property that the sum of pay-offs is zero. The only options are to draw (0, 0) or for one player to win and the other to lose (*x*, −*x*). Examples of strictly competitive games include the MPG (figure 1*a*) and RSPG (figure 1*b*). In the MPG, row and column players simultaneously choose heads (H) or tails (T). If they match, the row player wins (+1) and the column player loses (−1). If they do not match, the row player loses and the column player wins. The RSPG is similar except now players simultaneously choose rock (R), scissors (S) or paper (P). If they choose the same, they tie (0), but otherwise rock wins over scissors, scissors wins over paper and paper wins over rock. Optimal strategies involve randomization (or unanticipated play) resulting in uncertainty as to who will actually win. We assume that nature will favour players who can find any inherent advantage in these games, and therefore the brains of these players will be designed to find and exploit these advantages. One well-known system for exploiting advantages is through reinforcement learning (Sutton & Barto 1998).

In the ISG, the row player (acting as an employee) must decide whether to work (W) or shirk (S) (figure 1*c*). If the employee works, he gains 1 no matter what. But work is costly to the employee, who prefers to shirk and gain 2, unless the employee gets caught by the boss and gets 0. The column player, acting as the boss, must decide to inspect (I), the only way to detect shirking, or the boss could choose not to inspect (DI). The cost of inspection is −C. If the boss inspects (I) when the employee works, the boss gains 2−C, but if the boss inspects when the employee shirks the boss loses −C. However, if the boss fails to inspect (DI) when the employee shirks, the boss loses −2, but if the boss chooses not to inspect and the employee works, the boss gains 2. As in the strictly competitive games above, the optimal strategies for the employee and the boss will involve randomization.

In the PDG, two players must simultaneously decide to either cooperate or defect (Axelrod 1984; figure 1*d*). If they both cooperate, they do better, with a pay-off (2, 2) compared with (1, 1) when they both defect. However, each player's optimal strategy is to always defect and get 3 if the other player cooperates and avoid getting 0 if the other player defects.

The ESG, UG, DG, IG and TG in figure 2 are presented as extensive form games; each player has to *sequentially* make a choice at each of the decision nodes, *n*^i^, assigned to that player. The strategy sets, *S*_1_ and *S*_2_, now consist of all the different combinations of choices that a player can make. It is still reasonable to think of the strategy profile (*s*_1_, *s*_2_) as being chosen simultaneously, but in making these choices players will take into account the sequential order of the moves. In an extensive form game, a strategy profile picks a path from an initial decision node through the game tree to a terminal node {*t*^1^, …, *t*^*k*^}, where *k* is the number of different outcomes in the game. Similar to the strategic form games, players have preferences over the outcomes in the extensive form games, or equivalently over the strategy profiles that produce these outcomes.

In the ESG, player 1 is assigned to the decision node *n*_1_ and player 2 to the decision nodes *n*_2_ and *n*_3_ (figure 2*a*). Player 1 decides whether to offer player 2 an equal split of ($5, $5) or an unequal split of ($8, $2). Player 2 must then decide whether to accept or reject the offer. A play through the game tree is a connected path of branches through the decision nodes that end at one of the terminal (or outcome) nodes labelled as *t*^1^ through *t*^4^, with a resulting pay-off to player 1 of *U*_1_(*t*^*i*^) and to player 2 of *U*_2_(*t*^*i*^) for reaching the terminal node *t*^*i*^. For example, in the ESG, the choice of the branch labelled R (reject) by player 1 and the branch labelled A (accept) by player 2 results in the terminal node *t*^4^ and the pay-offs *U*_1_(*t*^4^)=8 and *U*_2_(*t*^4^)=2.

In the UG, player 1 must propose how to divide a fixed amount of money, say $10 (Güth *et al*. 1982; figure 2*b*). Once player 1 proposes, player 2 can either accept or reject. If player 2 rejects the proposal, both players earn zero; otherwise, the players earn the split proposed by player 1. The same logic applies to the ESG (figure 2*a*), but now the offers are restricted to either ($5, $5) or ($8, $2). Game theory predicts that player 2 should accept any positive offer, and player 1, reasoning this way, should offer player 2 some small amount, such as the proposal of ($8, $2) in the ESG.

Alternatively, the evolution of competitive instincts may cause player 2 to reject unequal offers in favour of more equal offers. While a rejection is costly, since both players get nothing, the threat of rejection often will improve a player's terms of trade (Hoffman *et al*. 1998). Further evidence for the evolution of inequity aversion has been reported by Brosnan & De Waal (2003) in their experiments with capuchin monkeys. Inequity aversion may lead to a willingness to engage in costly punishment (or negative reciprocity) in order to protect one from aggressive behaviour. Note, however, within the game is the implicit property right that allows player 2 to reject the offer without the threat of retaliation. While both players are clearly better off getting something, competitive instincts can cause them to get nothing. This suggests that an ability to deduce the mental state of the other person may help players calibrate their decisions to each other's mental state in order to avoid getting nothing (Frith & Frith 1999).

The DG was introduced as a means to control for the effects of punishment threats in the UG (figure 2*c*; Forsythe *et al*. 1994). In this game, a person is given $10 by the experimenter and is asked how he/she would like to divide the money between himself/herself and an anonymous person.

In the IG, two players are given some amount of money, say $10. Player 1 is then given the opportunity to send none, some or all of his/her $10 to player 2 (Berg *et al*. 1995; figure 2*d*). Whatever amount of money player 1 sends is increased by the experimenter by some amount, say tripled, e.g. if player 1 sends all $10, then player 2 will get $30. Player 2 then decides how much of the tripled money to send back to player 1.

The TG is a simpler version of the IG where player 1 is restricted to the option of sending nothing or sending all $10, and if the $10 is sent, then player 2 is restricted to either send nothing back or send half of the tripled amount, or $15, back (figure 2*e*). Game theory predicts that player 2 will send nothing back, and that player 1 will realize this and send nothing as well.

Alternatively, the evolution of reciprocity behaviours may cause the second player to feel a social obligation towards player 1's trust, i.e. by sending money. Note that the terms of exchange can be explicit, negotiated in a previous game, or implicit, established by social norms. Consequently, player 2's decision to cheat can be against either an explicit or implicit standard of reciprocity. While both players are clearly better off by extending trust and being willing to reciprocate, their competitive instincts can cause them to get nothing. This again suggests that an ability to deduce the mental state of the other person may help player 1's decisions when to trust and may help player 2's decisions when to reciprocate.

### (b) Game theoretic analysis of neuroeconomics games

Nash (1950) proposed a theory to predict player's behaviour in matrix games in terms of the Nash equilibrium (NE) of the game. A NE in pure strategies for a two-person matrix game is a strategy profile $\left(s_{1}^{*},s_{2}^{*}\right)$ that satisfies the following conditions:

(1.1) $$U_{1}(s_{1}^{*},s_{2}^{*})\geq U_{1}(s_{1},s_{2}^{*})\;\text{for}\;\text{all}\;s_{1}\in S_{1}$$

and

(1.2) $$U_{2}(s_{1}^{*},s_{2}^{*})\geq U_{2}(s_{1}^{*},s_{2})\;\text{for}\;\text{all}\;s_{2}\in S_{2}.$$

It is easy to verify that $s_{1}^{*}=\text{defect}$ and $s_{2}^{*}=\text{defect}$ is the only NE in pure strategies for the PDG (figure 1*d*). Furthermore, there are no pure strategy profiles that satisfy (i) and (ii) for the ISG or the MPG.

Nash (1950) recognized this problem and extended his equilibrium concept to allow for mixed strategies, or probability distributions over *S*_1_ and *S*_2_. In two-person games, with two choices each, we can define a mixed strategy as follows. Let Δ*S*_1_={(*p*^1^, *p*^2^)≥0: *p*^1^+*p*^2^=1}, where we interpret *p*^1^ as the probability of playing the pure row strategy *r*^1^, and *p*^2^ as the probability of playing the pure row strategy *r*^2^, then *p*∈Δ*S*_1_ is a mixed strategy for player 1 and, similarly, *q*∈Δ*S*_2_={(*q*^1^, *q*^2^)≥0: *q*^1^+*q*^2^=1} is a mixed strategy for player 2. Given a mixed strategy profile (*p*, *q*), we can define the expected utility for player 1 as follows:

(1.3) $$EU_{1}(p,q)=p^{1}q^{1}U_{1}(r^{1},c^{1})+p^{1}q^{2}U_{1}(r^{1},c^{2})+p^{2}q^{1}U_{1}(r^{2},c^{1})+p^{2}q^{2}U_{1}(r^{2},c^{2}).$$

Similarly, the expected utility for player 2 is as follows:

(1.4) $$EU_{2}(p,q)=p^{1}q^{1}U_{2}(r^{1},c^{1})+p^{1}q^{2}U_{2}(r^{1},c^{2})+p^{2}q^{1}U_{2}(r^{2},c^{1})+p^{2}q^{2}U_{2}(r^{2},c^{2}).$$

A NE in mixed strategies for a two-person game, with each person having two pure strategies, is a mixed strategy profile (p^*^, q^*^) that satisfies the following conditions:

(1.5) $$EU_{1}(p^{*},q^{*})\geq EU_{1}(p,q^{*})\;\text{ for}\;\text{all}\;p\in \Delta S_{1}$$

and

(1.6) $$EU_{2}(p^{*},q^{*})\geq EU_{2}(p^{*},q)\;\text{ for}\;\text{all}\;q\in \Delta S_{2}.$$

Note that a pure strategy NE is a mixed strategy NE with all the probability weight on one of the pure strategies. Nash (1950) proved that every strategic game has a NE in mixed strategies and that every pure strategy, which has a positive weight in a mixed strategy NE, must have the same expected utility for the player, for example *EU*_1_(*W*, q^*^)=*EU*_1_(*S*, q^*^). Using this result, it is easy to solve for p^*^=(1−*C*, *C*) and q^*^=(0.5, 0.5) for the ISG, with 0<C<1, p^*^=(0.5, 0.5) and q^*^=(0.5, 0.5) for the MPG, and p^*^=(1/3, 1/3, 1/3) and q^*^=(1/3, 1/3, 1/3) for the RSPG.

We can now write the pure strategy choices for a player in an extensive form game as a choice of branch at each of his/her decision nodes. So, for example, in the ESG, player 1 has the strategy set *S*_1_={*E*, ∼*E*} while player 2 has the strategy set *S*_2_={(*A*, *a*), (*A*, *r*), (*R*, *a*) and (*R*, *r*)}, where the first element of each pair is the choice made at *n*_2_ and the second element is the choice made at *n*_3_. We can define a mixed strategy profile (*p*, *q*) and a NE profile (p^*^, q^*^) as before. Note that, for the ESG, there are a number of NE profiles including (*E*, (*A*, *a*), (*E*, (*A*, *r*)) and (∼*E*, (*R*, *a*)). Selten (1975) offered as refinement of NE the subgame perfect Nash equilibrium (or SPNE) of the game. In the game shown in figure 2, we can find the SPNE of the game by using backward induction. So, for example, in the ESG, we can start with the nodes *n*_2_ and *n*_3_ and ask what player 2’s optimal choice is at each. The answer is for player 2 to always accept or (*A*, *a*). But given this choice, player 1 should now play E resulting in the SPNE profile (*E*, (*A*, *a*)). A similar unique SPNE profile holds for the UG, resulting in player 1 asking and getting 10−*e*, where e is the smallest divisible monetary unit.

If a game is repeated a number of times, this can lead to new strategies by players, in particular, where the strategy chosen for the current game is made conditional on previous plays of the game. For example, in the PDG, the tit-for-tat strategy studied extensively by Axelrod (1984) has a player who chooses to cooperate in the first play of the game and then in every play thereafter it has the player play whatever his/her opponent played in the previous play. While tit-for-tat is not a NE for a finitely repeated PDG, it can (when played against similar conditional strategies) result in better pay-offs for long periods of play than simply defecting in each period.

### (c) Experimental design for the measurement and control of expected utility

Any game can be turned into an experiment by assigning subjects (either human or non-humans) to the role of one of the players in the game. Subjects make decisions, either by choosing strategies or by choosing an action at each decision node in these roles. Finally, subjects are incentivized by paying them a salient reward for each outcome reached. Treatment conditions can then be varied to observe their effect on strategy choices. There are two important concerns in designing a game theory experiment: the first involves the ability to control or measure individuals' subjective values over outcomes in the game, and the second is the ability to control or account for the expansion of the strategy space due to repeated play of the game.

Generally, human subjects are incentivized with a monetary pay-off at each outcome while animals are incentivized with food (or juice) pay-offs. This allows the experimenter to induce a utility function over outcomes that is sufficient for studying pure strategy choices and pure strategy NE. This induction procedure assumes that subjects prefer more of the pay-off to less and that their decision costs of making a choice is low relatively to reward (Smith 1976). If such an assumption is suspected, pay-offs can be increased, through, for example, food deprivation in animal experiments, and/or explicit models of decision cost can be introduced to examine the data (e.g. Smith & Walker 1993).

When we consider mixed strategy NE, it is no longer certain that paying money induces the right preferences, since expected utility is a probabilistic weighting of the subject's true utility function. We can solve this problem either by estimating a subject's underlying utility function (Holt & Laury 2005) or by inducing such preferences by paying subjects in probability using the lottery procedure (Berg *et al*. 1986). Both approaches can lead to problems. Estimating preferences makes additional assumptions, which are routinely violated in experiments and can lead to very different estimates of expected utility based on the elicitation technique used (Berg *et al*. 2005). Inducing preferences also makes additional assumptions as it requires subjects to make calculations that are independent from their preferences, thus changing the nature of their decision and increasing their decision costs.

## 2. Primates playing economic games

While many species of animals have been studied in terms of games, the closest evolutionarily to humans are the other primates. First, we consider some of the single-cell firing studies that have been conducted while primates played games against a computer, and then we consider some of the experimental studies of primate behaviour in games with other primates.

### (a) Primate behaviour in games against a computer

Lee *et al*. (2004) examined the strategy choices of rhesus monkeys while playing a computer opponent in the MPG. The computer played three different strategies. Strategy 1 played the NE strategy (1/2, 1/2), making the monkey indifferent between playing H or T. Strategy 2 examined the monkeys play to see whether it could exploit any bias towards one of the choices, and if so exploited that bias. Strategy 3 extended strategy 2 by looking for serial correlation in the monkeys' choices and exploiting this bias as well. When the monkeys played against strategy 1, they tended to be biased towards one of the target choices and did not play the NE strategy. When the monkeys played strategy 2 they adjusted their strategies to play 50–50 as predicted by NE, but their choices were serially correlated as they tended to stick with a winning choice and switch from a losing choice. Finally, when the monkeys played strategy 3 they again adjusted their strategies to become less serially correlated and thus predictable. In a follow-up experiment, monkeys played the RSPG against a computer again following the three strategies (Lee 2005). Again, the monkeys adjusted their strategies to the strategy of the computer, although this time when playing against strategy 2, the monkeys tended to best respond to the computer's last play of the game.

Lee and his colleagues found that neurons in the dorsal lateral prefrontal cortex (DLPFC) and the anterior cingulate cortex (ACC) were involved in encoding strategic choices (Barraclough *et al*. 2004; Seo & Lee 2007). The authors found that a reinforcement learning model predicted the monkeys' behaviour, but the adaptive feature of the monkeys' responses to the different computer strategies was not explained by this model. Soltani *et al*. (2006) provided a neuronal model of monkey choices for the MPG. Their model adapts Wang's (2002) attractor network model where recurrent excitation within a local population of neurons together with an inhibitory network between populations can implement a ramping-to-threshold decision process. Within their model, Soltani *et al*. (2006) found that they can capture the adaptive changes in strategy choices if they include a belief-dependent learning rule that updates the synaptic strengths of neurons selecting for both chosen and unchosen actions. The demonstration of a biologically feasible computational process that can explain the functional choices of monkeys brings us one step closer to understanding how strategies can be learned, but it remains to be seen if the modelling in this case can be generalized to games with many or even a continuum of strategies, or to strategies in extensive form games. In particular, belief learning may have to be combined with more explicit accounts of decision costs.

Dorris & Glimcher (2004) examined the behaviour of monkeys and humans playing the ISG as they vary inspection costs and thus the mixed strategy of the worker. They found very similar behaviour in humans who played other humans, in humans playing a computer strategy (similar to strategy 2 in Lee *et al*. 2004) and in monkeys playing the same computer strategy. In every treatment, subjects’ average 20 trial plays were predicted by the mixed strategy NE for inspection costs of 0.5 and above, but workers (monkeys and humans) tended to shirk above the equilibrium prediction when inspection costs were 0.4 and lower. However, in examining neurons in the anterior parietal cortex (LIP area) of the monkeys using a choice design similar to Platt & Glimcher (1999), they found that the average firing rates of these neurons encode the relative expected desirability of the choices to shirk versus the choice to work. The authors conclude that even when subjects’ strategies deviated from the NE prediction they still played according to the relative expected utility calculations in LIP, which they now call physiological expected utility.

### (b) Primate behaviour in games with other primates

A number of behavioural experiments involving exchange type games have been run with monkeys. De Waal (1997) found that capuchins would engage in reciprocal sharing through facilitated taking in a delayed exchange task where monkeys had alternate access to a source of food. In a related task, de Waal & Berger (2000) showed that capuchins will increase facilitated taking when they have been helped by another monkey to acquire food in a cooperative bar pull. When capuchin monkeys are allowed to trade tokens for food, Brosnan & De Waal (2003) demonstrated that monkeys will reject an exchange with the experimenter after they observe another monkey getting a better deal. Recently, Jensen *et al*. (2007) looked at chimpanzees’ strategies in the ESG. The authors found that monkey responders accepted any offers no matter how unequal; however, monkey proposers did not take maximal advantage of the responders' strategies. Why did not chimpanzees show inequity aversion in the ESG? Brosnan & de Waal (2004) argued that monkeys housed together for 30 years do not exhibit inequity aversion, in the trade for tokens task, and it may be that such long-term groups have worked out repeated game strategies that no longer require inequity responses. Overall, these results suggest that monkeys will choose reciprocal strategies to improve cooperative gains; while monkeys show inequity aversion, it is less clear whether monkeys will act on inequity aversion in bargaining games and this may depend on whether or not they have had time to develop alternative repeat game strategies.

## 3. Humans playing economic games

Experiments on human subjects have focused on various two-person forms of classic social dilemma games involving the conflict between self-interest and mutual interest. The most well-known social dilemma is the PDG, but from an economics perspective, more interesting social dilemma games are those that capture human exchange such as the UG, IG and TG. In studying human exchange, it is important to have a control condition, the DG, which can help sort out the role of sympathy as compared with reciprocity.

### (a) Human behaviour in the Prisoner's dilemma game and its neural correlates

The PDG has been studied extensively with experiments (Axelrod 1984, 1997) which report on experiments where subjects submitted strategies to play other subjects’ strategies in a repeated PD game, and report on subsequent agent-based models of PD strategies. In his tournaments, Axelrod (1984) found that a simple strategy called tit-for-tat (submitted by Rapoport & Chammah 1965) finished first. This strategy was very simple; it starts by cooperating and then mimics whatever its opponent did in the previous period. Note that the worst-case scenario, if tit-for-tat plays the always defect strategy, is the (C, D) pay-off in the first period, but this is more than offset by tit-for-tat's ability to cooperate, and reach (C, C), with other cooperative strategies. More recently, Bo (2005) has studied the subjects' behaviour under more controlled conditions in both one-shot and repeated play lasting either a finite number of plays (finite horizon games), or when there was a fixed probability that play will end on a given round (infinite horizon games). The author found that subjects cooperated only 10 per cent of the time in a one-shot PDG, but first-period cooperation increased to roughly 35 per cent in games lasting four plays, but fell back to 10 per cent by the fourth play. Finally, first-period cooperation started at 46 per cent in games with a 3/4 chance of continued play, and stayed statistically higher (approx. 30%) for all periods compared with the 10 per cent rate found in one-shot or cooperative rates of play in the last period of finite horizon games.

Neuroeconomics and social neuroscience experiments have begun to study some of the neural underpinnings of economic game playing. Using fMRI, Rilling *et al*. (2002) scanned the brains of women who played other women (or a computer) for at least 20 rounds of a PDG. Subjects who experienced continued (C, C) outcomes showed higher activation in the ventral striatum, but subjects who played cooperate also showed a decrease in ventral striatum activity when they saw that their opponent played defect. Activity in the ventral striatum is consistent with a reinforcement learning model where subjects expect that cooperation will result in the higher (C, C) pay-off. Orbitofrontal cortex activity was correlated with the evaluation of outcomes by post-scan interviews with peak magnitudes of fitted BOLD responses the highest at CC outcomes, reported by subjects as most desirable, next highest at CD outcomes, third highest at DC outcomes and the lowest fit DD outcomes, reported by subjects as least desirable. Finally, activation in the rostral ACC was more active when subjects choose to cooperate after their partner cooperated; this is consistent with the role of the ACC in the detection of cognitive conflict (Carter *et al*. 2000). In a follow-up experiment by Rilling *et al*. (2004*b*), subjects played repeated one-shot PDGs with different counterparts. The fMRI experiment replicated the earlier findings and strengthened the conclusion that subjects in repeat PDGs learn to cooperate by using neural-based reinforcement learning strategies. Finally, Rilling *et al*. (2007) have also looked at the effect of (C, D) outcomes on subjects who played cooperatively only to be defected on. The authors find that male subjects who scored high on the Levenson total psychopathy test showed less amygdala activation when encountering a (C, D) outcome, and were less cooperative over all. It may be that amygdala responses to (C, D) cause subjects to avoid behaviours that can lead to this outcome by either defecting against a cooperative partner (and thus inviting retaliation) or cooperating against a non-cooperative partner.

In addition, a number of pharmacological interventions have been studied in subjects playing PDG- or PD-like games. For example, Tse & Bond (2002*a*) found that a single dose of reboxetine, a noradrenaline reuptake inhibitor that elevates the extracellular concentration of the neurotransmitter noradrenaline, resulted in more cooperative play in subjects playing a repeated PD-like game. In a follow-up study, Tse & Bond (2002*b*) looked at the effects of a two-week treatment with citalopram, a serotonin reuptake inhibitor, which also resulted in subjects playing more cooperatively in a PD-like game. Moreover, Wood *et al*. (2006) found that subjects deprived of l-tryptophan, and thus having lower serotonin levels in the brain, were less cooperative in a repeated PDG than subjects who were not. However, lower cooperation was only found on the first day of a two-day study, suggesting that subjects who are l-tryptophan depleted will find ways to adjust back to a more cooperative strategy.

### (b) Human behaviour in the dictator game and its neural correlates

In the DG, a person is given $10 by the experimenter and is asked how he/she would like to divide the money between himself/herself and an anonymous person (Forsythe *et al*. 1994). When this game is run as an experiment only 21 per cent of the subjects kept all the money. The money that is sent is sometimes seen as a measure of altruism, or at least sympathy, that subjects have to one another. Alternatively, Hoffman *et al*. (1996) hypothesized that our evolved social brain would be sensitive to the likelihood of being seen as and/or found out to be non-cooperative based on one's group behaviour. The authors called the inverse of this likelihood ‘social distance’ and hypothesized that the greater the social distance, the lower the likelihood of being typed as non-cooperative, and the more likely a subject will keep all the money. In their double-blind experiment, designed to maximize social distance, subjects were much more self-interested with a majority, 64 per cent, keeping all the money.

Both the Forsythe *et al*. (1994) and the Hoffman *et al*. (1996) experiments can be criticized owing to the use of ‘house money’. Cherry *et al*. (2002) examined dictator's giving when the dictator had to earn their money in the experiment, before deciding how much to send. Using a double-blind control, they found that 95 per cent of the dictators kept the money, suggesting that a subject's sense of ownership or entitlement to the money will affect how much they will give. In social psychology, equity theory can be used to explain the results from these and many similar experiments (Adams 1965). Equity theory assumes that subjects in an experiment assign subjective weights to the contribution of themselves and others and expect to receive earnings for themselves, and for other subjects, to be proportional to their relative contributions. When subjects fail to earn what they expect, or they see other subjects failing to earn what they expect those subjects should get, this can cause a negative emotion, which subjects may try to avoid or which may motivate corrective action. When using house money, subjects may view showing up for the experiment as the major contribution, while earning money may shift subjects' views to contributions made in the experiment.

In a recent fMRI experiment, Spitzer *et al*. (2007) investigated brain activities of dictators when there was a possibility that they could be punished by recipients compared with dictators who were safe from punishment. Subjects were given an additional amount of money each round, but, in the punishment condition, recipients were told they could either keep this money or use it to reduce their dictator's earning. In particular, one unit of money spent by the recipient reduces the dictator's earning by five units. The threat of punishment effectively provides an immediate consequence to getting caught, causing dictators to be significantly more generous. The authors used the Machiavelli instrument (Christie & Geis 1970) to predict how selfish subjects will be towards others. They found that subjects with high Mach scores showed the greatest transfer differences between conditions. Furthermore, the higher the subject's Mach score was, the greater was the amount of insula activity. Finally, the right DLPFC and right caudate nucleus showed increased brain activity as the average transfer difference between conditions became larger. It would be interesting to see how these results change when dictators are able to earn their money.

### (c) Human behaviour in the ultimatum game and its neural correlates

When the UG was first run with cash-motivated subjects, Güth *et al*. (1982) observed that the modal proposal was to split the money 50–50. This result has been replicated dozens of times. For example, Forsythe *et al*. (1994) compared offers in the UG with those in the DG and showed that the 50–50 proposals in the UG are largely a consequence of player 2's ability to reject player 1's proposal. Thus, to reduce the risk of rejection, player 1 makes more conciliatory offers.

Hoffman *et al*. (1994) tested the predictions of social exchange theory in the UG. The authors included two social exchange conditions. A contest in which subjects earned the right to be player 1 and a socially defined seller/buyer exchange roles for players 1 and 2 were compared with a baseline condition with random assignment to the first player position and neutral role definitions. In the baseline condition, half of the offers were at $5 with a mean offer to player 2 of $4.37. By comparison, the property right assignments with buyer/seller roles resulted in less than 10 per cent of the offers at 50–50 with a mean offer to player 2 of $3.08, which was predicted by authors to have the strongest equity norm effect. In both cases, rejection rates were low, at approximately 10 per cent, suggesting that first players' low offers were no more risky. This suggests further that second players implicitly recognized the right of their counterpart to offer less when they had earned the right to do so.

In an fMRI study, McCabe *et al*. (2001) studied brain activation in humans who played sequential two-person simplified UGs and TGs for cash rewards. Half of the time, subjects played as player 1, the other half as player 2. Each time they played, their counterpart was either a computer playing a fixed probabilistic strategy or a human who was recruited to play outside the scanner. Subjects were told for each play whether they were playing the computer or the human. The authors conjectured that subjects would use mentalizing (Frith & Frith 1999) to infer the intentions of the other player. Mentalizing would play an important role in the binding of mutual pay-off information to a cooperative event representation and thus invoke cognitively strategies for delay of gratification, and therefore produce trust and reciprocity. Based on their individual plays, seven out of the 12 subjects were labelled as cooperators while five were labelled as non-cooperators. In a conjunction analysis, the seven cooperators all showed greater activations in the anterior region of the rostral medial prefrontal cortex (arMFC). Recent research has shown that the arMFC is not only involved in representing our own thoughts, feelings and beliefs, but also in representing the mental states of other people, and is activated in a variety of social cognition tasks such as self-knowledge, person perception and mentalizing (for a review see Amodio & Frith 2006). The authors argue that the observed activation in cooperators is consistent with shared reciprocity intentions, resulting in the inhibition of both individual reward seeking by player 2 and risk-avoiding behaviour by player 1.

In another fMRI study, Sanfey *et al*. (2003) investigated the neural correlates of the second player's behaviour in the UG. Subjects made 20 decisions, while playing 10 games with other individuals and 10 games with the computer. In the human counterpart condition, subjects were told they would play once against each of 10 different humans, but, in fact, the experimenters determined the sequence of offers subjects would face to ensure that the human and computer offers were counterbalanced between five 50–50 (fair), one 70–30 (less fair), two 80–20 (unfair) and two 90–10 (unfair) offers. Behaviourally, subjects accepted all fair and most of the less-fair offers, however rejected roughly 50 per cent of the unfair offers by humans, while accepting roughly 80 per cent of the unfair offers by the computer. The authors found by contrasting unfair with fair offer activations in the ACC, bilateral DLPFC and bilateral anterior insula (AI). Specifically, as the activation of right AI increased, the more probably a subject rejected an unfair offer. The authors argue that the ACC activation reflects the motivational conflict between fairness and self-interest when facing unfair offers, the AI activation the degree of emotional resentment of unfair offers and the DLPFC the cognitive control of the emotional impulse to reject unfair offers. Importantly, Knoch *et al*. (2006*a*) found in a subsequent study that low-frequency TMS of the right DLPFC, but not of the left DLPFC, increased the acceptance rate of unfair offers relative to a placebo stimulation (from 9 to 44%). The authors concluded that the right DLPFC is not critical in controlling the impulse to reject unfair offers. It may be that DLPFC instead represents offers as fair and unfair and that subjects with impaired DLPFC simply accepted all offers.

Another fMRI study by Tabibnia *et al*. (2008) investigated the neural correlates of the recipient's behaviour in the UG and found AI activation during rejected trials. In addition, the authors found activation in the right VLPFC (relative to a resting baseline) when unfair offers were accepted, indicating that this region might regulate the resentment associated with unfair offers down.

The Sanfey *et al*. (2003) experiment resulted in a follow-up behavioural study by Xiao & Houser (2005), who investigated the emotional expressions of subjects in the UG and found that subjects (second players) who can express anger (or disgust) to their counterpart (first players) for an unfair offer are significantly more likely to then accept the offer.

### (d) Human behaviour in the investment/trust game and its neural correlates

Berg *et al*. (1995) gave two players $10 as a show-up fee in a double-blind IG. Player 1 was then given the opportunity to send none, some or all of his/her $10 to player 2. Whatever amount of money was sent was tripled, e.g. if player 1 sent all $10, then player 2 would get $30. Player 2 then decided how much of the tripled money to send back to player 1. The subgame perfect equilibrium prediction is that player 2 should keep all the money, and therefore player 1 should send nothing. Alternatively, social norms may exist that interpret sending money as an obligation for player 2 to reciprocate. All but two of the 32 players sent some amount of money to the other player, with two-thirds sending $5 or more, and about half of these high-trust subjects got more sent back to them than they originally sent before tripling.

McCabe & Smith (2000) introduced the TG as a simplified form of the IG. The game has only two choices for each player. Player 1 can choose to end the game by moving left, giving each player $10, or choose to continue the game. If player 1 chose to continue, player 2 can choose between either player 1 gets $15 and player 2 gets $25 or player 1 gets $0 and player 2 gets $40. The choice made by player 1 is risky and can be interpreted as trusting player 2, since player 1 gave up $10 and might end up with $0. Similarly, the decision by player 2 to choose that player 1 gets $15 and player 2 gets $25 can be interpreted as being trustworthy since player 2 gave up $40 and only received $25. When played as a one-shot game, half of the player 1's were trusting and three-quarters of the player 2's, who then get to move, were trustworthy.

To test whether mentalizing may be important in playing the TGs, McCabe *et al*. (2003) compared behaviour in the standard TG with behaviour in an involuntary TG where player 1 is forced to move down and player 2 was informed about it. The authors found that player 2 is twice as likely to make the trustworthy decision in the TG compared with player 2 in the involuntary TG. They argue that the increased propensity to reciprocate player 1's trust in the TG occurred because player 2 inferred player 1's intentions to cooperate since player 1 has given up a sure thing, i.e. $10, to make them both better off. This inference is what leads to a greater trustworthiness.

Repetition of the IG allows players to form a reputation with respect to a behavioural type (such as being a trusting or trustworthy individual). Using a multiround version of the IG, King-Casas *et al*. (2005) found that responses in the dorsal striatum (head of caudate nucleus) of player 2 were greatest when player 1 invested more in response to player 2's previous reciprocity. In addition, player 2's intention to reciprocate was observed as a shift in peak activity in the caudate nucleus from the time when player 2 saw player 1's decision to before player 1's decision, suggesting that player 2 learnt to anticipate player 1's trustworthiness. It has been proposed that the caudate nucleus may serve as a key component of an ‘actor–critic’ model processing the contingent behaviour that led to the feedback, with the purpose of guiding future behaviour (O'Doherty *et al*. 2004; Tricomi *et al*. 2004). Furthermore, Tomlin *et al*. (2006) applied fMRI to scan two subjects' brains simultaneously as they played repeated interaction IG. These joint brain measurements showed agent-specific responses along the cingulate cortex for encoding decisions of other and oneself independently on metrical aspect of the economic exchange.

In another iterated version of the IG, Delgado *et al*. (2005) investigated whether prior social and moral information about potential trading partners affects this aforementioned neural reward circuit. Subjects were involved in two-person interactions and asked to make risky decisions about whether to trust fictitious trading partners after they received vivid descriptions of life events that indicated either their neutral, praiseworthy or suspect moral character. Although all three fictitious partners repaid in the IG with the same frequency, the caudate nucleus activated more strongly for repayment outcomes from the neutral partner, but not from the other partners. The authors argue that prior moral beliefs can influence economic decision making. Since the neutral partner represents unpredictable outcomes and there is more to learn, the human caudate nucleus presumably influenced the adjustment of choices based on feedback mechanisms in the neural circuitry of trial-and-error reward learning.

Furthermore, de Quervain *et al*. (2004) investigated the neural basis of altruistic punishment of defectors in the context of the IG. Using PET, subject's brains were scanned while they learnt about the defector's abuse of trust and determined the punishment. This experiment demonstrated that the dorsal striatum (caudate nucleus) was activated in the contrast between effective punishment (reduction of the defector's pay-off) and symbolic punishment (non-reduction of the defector's pay-off). Subjects with stronger activations in the dorsal striatum were willing to incur greater costs to punish. The authors argue that individuals derive satisfaction from punishing norm violations and the anticipated satisfaction from punishing defectors is reflected in the dorsal striatum activations.

Krueger *et al*. (2007) investigated the neural correlates of trust combining fMRI with a non-anonymous repeated TG. The authors showed that two different brain systems may be used to develop the first player's trust. A personal ‘unconditional’ trust system involved early activation of the arMFC (mentalizing) followed by later activation of the septal area, a limbic region that has been demonstrated to modulate various aspects of social behaviour such as social attachment (Numan 2000) by controlling anterior hypothalamic functions and the release of the neuropeptides vasopressin and oxytocin (Powell & Rorie 1967; Loup *et al*. 1991; Insel & Young 2001). Besides the well-known physiological functions of oxytocin in milk let-down and during labour, oxytocin is a key mediator in facilitating various complex social behaviours, including maternal care (Insel & Young 2001), pair bonding (Insel & Shapiro 1992) and social recognition (Choleris *et al*. 2003) in animals and social attachment (Bartels & Zeki 2004; Aron *et al*. 2005), generosity (Zak *et al*. 2007) and interpersonal trust (Zak *et al*. 2005) in humans. The authors argue that repeated experience with another player's cooperation can lead to the evaluation of that player as a ‘trustworthy’ person, resulting in an increased production of oxytocin and allowing greater trust. A second ‘conditional’ trust system seems to be more situational and less personal. This system does not use the mentalizing system early on but does use the reinforcement learning system (ventral tegmental area) to build trust. In brains using this system, mentalizing activation was observed in the latter stages of play, but not in early play, suggesting that situational trust uses the mentalizing system to fine-tune expectations over when a counterpart will defect.

There is recent evidence that greater first player's trust can be induced in strangers by the intranasal administration of oxytocin during interpersonal exchange. Kosfeld *et al*. (2005) showed that the effect of oxytocin on trust is not due to a general increase in the readiness to bear risks, but it specifically affects an individual's willingness to accept social risks arising through interpersonal interactions. In a follow-up fMRI experiment, Baumgartner *et al*. (2008) found that subjects who were given synthetic oxytocin intranasally showed no change in their trusting behaviour after they learned that their trust has been betrayed several times. Differences in trust adaptation were associated with reduction in activation in neural systems mediating fear processing (amygdala and midbrain regions) and adaptation to feedback information (dorsal striatum).

## 4. Trust and reciprocity in humans: an fMRI investigation

Previous analyses of the IG and TG games indicate the importance of different systems such as mentalizing, reward systems and social attachment for the neurobiology of trusting and reciprocity. However, little attempt has been made so far to identify the differences of underlying neural architecture for trusting and reciprocating behaviour. We present here additional analyses of the repeated TG experiment studied in Krueger *et al*. (2007). In this experiment, two strangers of the same sex—each in a separate MRI scanner—interacted with one another in a sequential reciprocal TG while their brains were simultaneously scanned (figure 3*a*). Subjects were asked to make sequential decisions for monetary pay-offs (low, medium or high in cents) presented in a binary game tree (figure 3*b*). Player 1 can either quit the game by not trusting player 2, resulting in a small equal pay-off for both, or player 1 can continue the game by trusting player 2, hoping to receive a better pay-off. Player 2 can reciprocate the first player's trust, giving them both a higher pay-off, or defect on player 1's trust, resulting in an even higher pay-off for player 2 and a pay-off of zero for player 1. In the control games, partners followed the same timeline as in the TGs, but they did not have to interact with one another and merely had to choose between lower and higher monetary rewards. The design of our experiment allowed us to address the question of which brain regions modulate trust and reciprocity during economic exchange.

### (a) Shared networks for trust and reciprocity

Using a general linear model analysis, we first sought brain regions whose BOLD responses were commonly recruited for decisions to trust and reciprocate. We identified two regions, the arMFC and the AI, by performing a conjunction analysis between decisions to trust and reciprocate (figure 4*a*).

Converging evidence from neuroimaging over the last decade suggests that the arMFC plays a critical role in mentalizing, which is the ability to represent another person's psychological perspective (for a review see Amodio & Frith 2006). It has been shown that mentalizing is impaired in autism (Baron-Cohen *et al*. 1985), schizophrenia (Frith & Corcoran 1996) and cerebral lesions (Stone *et al*. 1998; Happe *et al*. 1999; Stuss *et al*. 2001). A wide range of different paradigms has shown consistently arMFC activation, ranging from off-line tasks such as story and cartoon comprehension as well as viewing of real-time interactions (Gallagher *et al*. 2000; Frith & Frith 2003; Saxe *et al*. 2004) to online tasks such as playing economic games (McCabe *et al*. 2001; Gallagher *et al*. 2002; Rilling *et al*. 2004*a*; Fukui *et al*. 2006; Krueger *et al*. 2007; Hampton *et al*. 2008). We argue that mentalizing allows both players to predict the behaviour of the other and to engage successfully in a cooperative interaction by recognizing that the other player has independent experiences, beliefs and intentions. Both players have to think about how the other player thinks about them, i.e. before they invest they have to decide not just whether they trust their partner, but also whether their partner will reciprocate their trust.

The AI region has been consistently associated with empathy, which plays a fundamental social role allowing the sharing of experience, feelings and goals across individuals (Preston & de Waal 2002). Two major roles for empathy have been proposed (de Vignemont & Singer 2006): an epistemological role to provide information about the future actions of other individuals and a social role to serve as the origin of the motivation for cooperative and pro-social behaviour. Recent neuroimaging studies have shown that individuals share the emotions of others, when exposed to their emotions by the observation or imagination, and they activated parts of the same neuronal network as if they would have processed the same state in themselves (Wicker *et al*. 2003; Keysers *et al*. 2004; Singer *et al*. 2004*b*; Jackson *et al*. 2005).

In a recent fMRI study, Singer *et al*. (2006) has shown that the empathic response can be modulated by the reputation of the person we are observing. Subjects first played repeated PDGs to create good and bad reputations for two previously unknown partners. Afterwards, the brain activity of those subjects was measured while they observed that their confederates received pain. An empathy-related activation was observed in the AI and ACC, when the cooperative player was in pain. On the contrary, less empathy-related activation was observed for partners who had acquired a bad reputation through defection in the game. In another study, Singer *et al*. (2004*a*) allowed subjects to face a series of cooperative and no-cooperative opponents in a sequential PDG. In a subsequent sex assessment fMRI task, the authors demonstrated that simply displaying the faces to these cooperative partners in contrast to neutral faces revealed activations in reward- and emotion-related areas such as striatum, amygdala and insula. This finding suggests that trustworthy persons' faces trigger emotion and reward expectation.

We argue that empathy provides a more precise and direct estimate of other people's future actions, since shared emotional networks also directly elicit the activation of associated relevant motivational and action systems (de Vignemont & Singer 2006). Both players are in a cooperative relationship and one knows that the other person is the source of one's own affective state. By sharing their empathy state, they also share their emotional and motivational states, enabling them to make faster and more accurate predictions about the partner's future actions.

### (b) Specific networks for trust and reciprocity

Because the psychology of trust is relevant for player 1, whereas the psychology of reciprocity is important for player 2, we next explored which brain regions were only involved in trusting behaviour and not in reciprocating behaviour and vice versa.

Trusting is always risky given the unpredictability of the intentions of the partner in a social exchange (Fehr & Fischbacher 2003). Decisions to trust compared with decisions to reciprocate revealed activations in the lateral frontopolar cortex (FPC) and the right temporoparietal junction (TPJ) (figure 4*b*). Accumulating neuroimaging evidence indicates that the right TPJ plays a critical role in social cognition such as perspective taking (Ruby & Decety 2003; Jackson *et al*. 2006*b*), sense of agency (Ruby & Decety 2001; Farrer & Frith 2002; Decety & Sommerville 2003; Farrer *et al*. 2003), empathy (Jackson *et al*. 2006*a*; Lamm *et al*. 2007) and mentalizing (Saxe & Wexler 2005; Lawrence *et al*. 2006). However, a recent fMRI study by Mitchell (2008) demonstrated that the activity in the right TPJ is not selective for mentalizing. Furthermore, Decety & Lamm (2007) demonstrated in a recent quantitative neuroimaging meta-analysis that the right TPJ is not domain specific to social cognition, but rather is a more lower level computational mechanism involved in generating, testing and correcting internal predictions about external sensory events (Blakemore & Frith 2003). We argue that mentalizing depends on the coordinated interaction of both domain-specific abilities represented in the PFC and domain-general abilities represented in the posterior cortex such as TPJ (Adolphs 2001; Decety & Grezes 2006). The TPJ helps us to simulate another person's experience and interpret their actions within the context of their current choice which is then decoupled from reality in the medial PFC to provide an abstract encoding of the intentions of another person.

The FPC encodes meta-cognitive representations that enable us to reflect on the long-term values linked to the outcomes of our decisions (Wood & Grafman 2003; Tanaka *et al*. 2004). A recent meta-analysis of the functional specialization within the FPC (Brodmann area 10) revealed a functional variation between lateral and medial subregions of the FPC (Gilbert *et al*. 2006). Mentalizing was more likely to be associated with activation in the caudal medial FPC, whereas activation in the lateral FPC supports high-level guidance of task performance over extended periods of time (Christoff & Gabrieli 2000). Humans often sacrifice material benefits to endorse or oppose societal causes based on moral beliefs, which has been the target of recent experimental economics studies (Moll *et al*. 2005). Recently, Moll *et al*. (2006) used fMRI to investigate charitable donation behaviour while participants anonymously donated to or opposed real charitable organizations related to major societal causes. The authors demonstrated that the more anterior sectors of the PFC were distinctively recruited when altruistic choices prevail over selfish material interests. We argue that to make an exchange, it is necessary that first players overcome the desire for immediate gratification in favour of greater but postponed gains from mutual cooperation. Based on such a mechanism, we are able to balance immediate motives against the long-term consequences of our choices and long-term benefits in real social interactions (Wood & Grafman 2003).

In sum, our findings extend previous knowledge of the neural basis of trust and reciprocity in two-person reciprocal exchange. Trusting and reciprocating behaviour draw upon common neural systems of mentalizing (arMFC and TPJ) and empathy (AI). Both mentalizing (cognitive sharing with another person) and empathy (affective sharing with another person) involve an ability to simultaneously distinguish between different possible perspectives during reciprocal exchange. In addition, trusting behaviour specifically recruited an evaluation system for prospective outcomes (FPC). This more recently evolved system provides a mechanism that enables individuals to weight long-term rewards over immediate short-term gains allowing, therefore, mutual cooperation. The interplay of these neural systems supports reciprocal exchange that operates beyond the immediate spheres of kinship, one of the distinguishing features of the human species.

## 5. Summary

In this paper, we reviewed the results from a number of game experiments that establish a unitary system for forming subjective expected utility maps in the brain, and acting on these maps to produce choices. Game playing in humans involves two major systems: a valuation-choice system for making trade-offs and a shared social system for understanding and sharing mental states. The valuation-choice system has been the target of numerous studies of both human and non-human subjects, resulting in a relatively unified model of decision making involving a reinforcement learning system that calculates the expected utility of different choices, an expected utility map that weights the relative value of different choices and an all or nothing competition to make the final choice. However, more work needs to be done to better understand how neuronal systems learn to construct the underlying decision problems, and how neuronal systems perform backward and forward induction in a multistage decision process such as those made in extensive form games. For example, it is not clear whether strategies themselves are choice variables in the brain or whether strategies are simply stable constructs of the choices made at decision nodes.

The shared social system has also been the target of numerous studies, but largely with human subjects. These studies suggest that human subjects use both empathy (shared affect) and mentalizing (shared intentions) to better understand other players in the game and that neural computations that allow shared mental states affect the way games are defined in the brain and, consequently, how experience in games is encoded. While empathy and mentalizing clearly affect the valuation-choice system, there does not exist a biologically plausible computational model of how this occurs.

Within the existing paradigms of single-cell recording studies in monkeys and neuroimaging studies in humans, more work needs to be done to develop games that both monkeys and humans can play. So far, little has been done along these lines, and the little that has been done has not controlled for differences in how monkeys or humans are trained or how they make their decisions. Yet, this is important to adequately define, and study, the homologous brain regions that are assumed to exist between monkeys and humans.

At the same time, more work needs to be done to develop better game controls for studying the different neuronal computations involved in game play. For example, Cox (2004) has reanalysed the IG using separate controls for other regarding preferences. He has found that some decisions typically labelled as trusting and trustworthy are mislabelled, but are due instead to subjects' altruism to their partners. It is also important to explicitly account for repeated game strategies by having subjects play finitely repeated games with the same partner but be repaired with a new partner after each sequence.

Furthermore, future theory and research need to gain a better understanding of how the dispositions and behaviours of players affect how they think, feel and behave in economic games. In addition, research should explore how and why certain combinations of partner attributes promote or impede the development of social preferences (Fehr & Camerer 2007). Nevertheless, for the social neuroscience and economics to advance, we must gain a deeper understanding on how social disorders such as autism, Asperger's and Williams' syndrome, social phobias and antisocial personality disorder are linked to differences in neural activation during economic exchange. For example, Sally & Hill (2006) compared the behaviour of healthy with autistic individuals in the UG and PDG. The authors showed that autistic individuals were more likely to accept initial low offers in the UG and had a more difficult time shifting strategy in the PDG. Moreover, comparing the behaviour of patients with focal brain lesions with healthy controls is also an important step in proofing the prerequisite of various brain regions for particular game behaviours. For example, Koenigs & Tranel (2007) employed the UG to investigate decision making in patients with ventromedial PFC (VMPFC) damage. The authors demonstrated that the VMPFC group showed a higher rejection rate for each of the most unfair offers, showing that the VMPFC is a critical brain region in normal economic decision making. Moreover, temporary brain lesions induced by TMS might be helpful to identify the neural processes involved in decisions in which standard economic models predict behaviour (e.g. Knoch *et al*. 2006*b*).

By working together within the formal construct of game theory to build experiments to study a positive theory of game play, neuroscientists and economists are beginning to develop new insights that benefit both disciplines (Sanfey 2007). One benefit for economists is that expected utility, the fundamental underpinning of game theory, is operating as observable phenomena in neurons. As a consequence, deviations from maximizing behaviour may not be due to failures of expected utility, but may more likely be due to how subjects construct their understanding of the game through both their own and their shared mental experience. A benefit for neuroscientists is the mathematical formalism that the theory of games puts on social decision making. In particular, this leads to the understanding that social strategies are not just functions of a single brain's computation, but also a function of extrinsic equilibrium conditions, which produce external computations, which end up shaping how the brain decides.

## Figures and Tables

**Figure 1:**
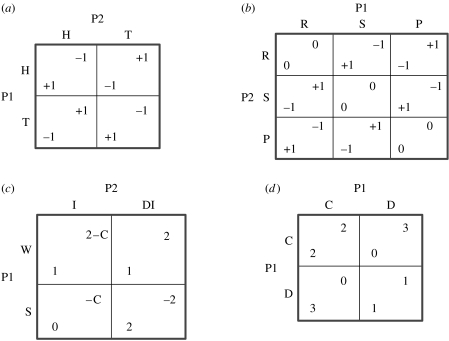
Matrix games: (*a*) matching pennies game (MPG: H, head; T, tail), (*b*) rock–scissor–paper game (RSPG: R, rock; S, scissor; P, paper), (*c*) inspection game (ISG: W, work; S, shirk; I, inspect; DI, do not inspect; C, cost of inspection), and (*d*) Prisoner's Dilemma game (PDG: C, cooperate; D, defect). P1, player 1; P2, player 2.

**Figure 2:**
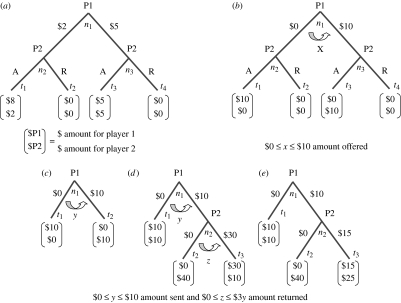
Bargaining and trust games: (*a*) equal split game (ESG), (*b*) ultimatum game (UG), (*c*) dictator game (DG), (*d*) investment game (IG), (*e*) trust game (TG). P1, player 1; P2, player 2; A, accept; R, reject; *n*_*i*_, decision nodes; *t*_*i*_, terminal nodes.

**Figure 3:**
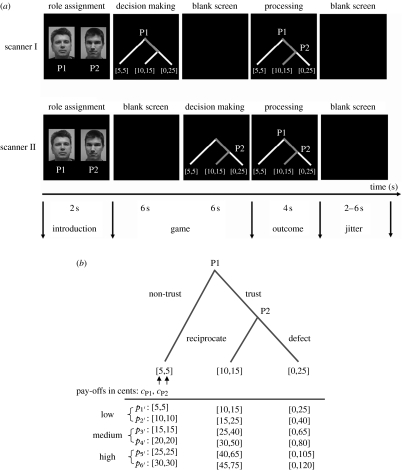
Experimental design. (*a*) Timeline for a single trust game. Partners were introduced by seeing each other via webcam and digital photographs were taken to be used for game trials. A 2 s introductory screen informed partners of the role that they were playing (P1 or P2). P1 saw the game tree, had to make a decision (non-trust or trust) within 6 s and waited 6 s for P2's decision while seeing a blank screen. P2 saw a blank screen for 6 s, saw the game tree with P1's decision and had to make a decision (reciprocate or defect) within 6 s. If P1 had chosen not to trust P2, the game was over and P2 saw P1's decision for 6 s. The partners saw the outcome of the game for 4 s followed by a blank screen with a jittered inter-stimulus interval of 2–6 s. (The partners played 36 voluntary trust games and 16 control games.) (*b*) Voluntary trust game. Partners made sequential decisions as first player (P1) and second player (P2) for pay-offs in cents (c: [c_P1_,c_P2_]) presented in a binary decision tree. P1 can choose left (non-trust) and quit the game with a small pay-off for P1 and P2 (e.g. [5,5]) or can choose right (trust) to continue the game. P2 can then choose left (reciprocate) giving them both a higher pay-off (e.g. [10,15]) or choose right (defect) resulting in an even higher pay-off to P2 and a pay-off of zero to P1 (e.g. [0,25]). Pay-offs (*p*_1′_–*p*_6′_) were split into three types: low (*p*_1′_–*p*_2′_); medium (*p*_3′_–*p*_4′_); and high (*p*_5′_–*p*_6′_).

**Figure 4:**
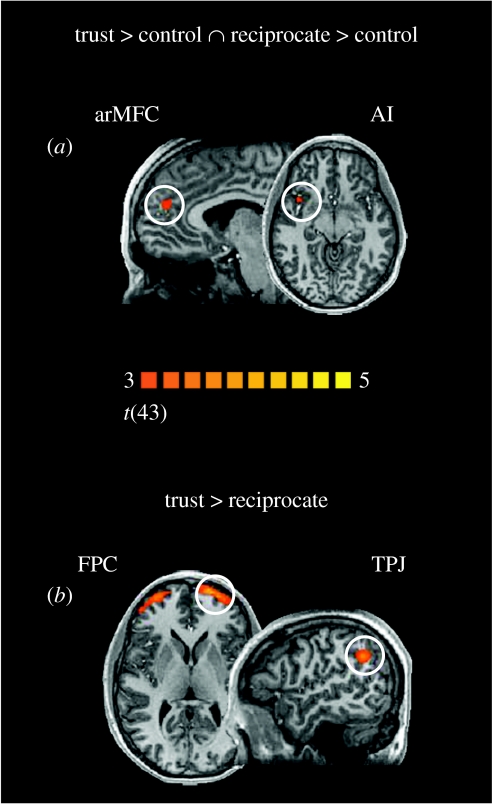
Brain responses. (*a*) Conjunction analysis. The anterior rostral medial prefrontal cortex (arMFC; BA 9/32; *x*, *y*, *z*; 6, 47, 22) and the anterior insula (AI; BA 13; 28, 15, −4) were commonly activated for trusting and reciprocating behaviour. (*b*) Trust versus reciprocate. Decisions to trust compared with decisions to reciprocate activated the bilateral frontopolar cortex (FPC; BA 10; −18, 62, 10; 30, 59, 7) and the temporoparietal junction (TPJ; BA 40; 48, −52, 34). Statistical images were superimposed on a template structural brain in Talairach space and thresholded at *p*<0.005, uncorrected, with an extent threshold of 100 mm^3^ (*t*=3.00, random effects). *A priori* regions hypothesized to be active were tested for activity using a small volume correction of a sphere of 10 mm and a false discovery rate (FDR) with a threshold of *q*(FDR)<0.05 (small volume corrected) and a cluster size threshold of 100 mm^3^.
